# Supplementary figures and images for: The oxidative stress response, in particular the katY gene, is temperature-regulated in Yersinia pseudotuberculosis
Source: PLoS Genet. 2023 Jul 10;19(7):e1010669. doi: 10.1371/journal.pgen.1010669 (PMC10358904; doi:10.1371/journal.pgen.1010669)

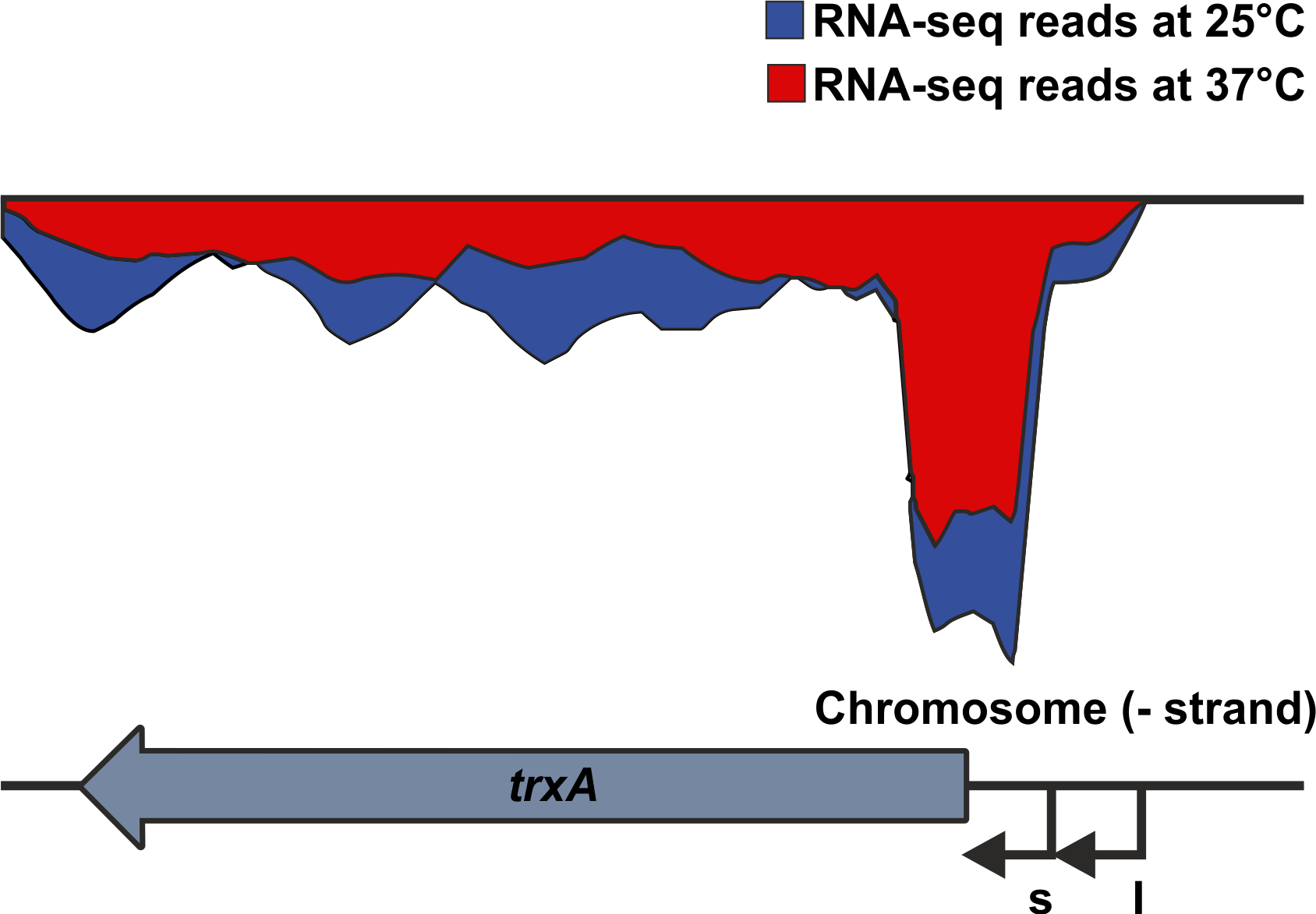

Supplement: S1 Fig — (TIF) [file pgen.1010669.s001.tif]

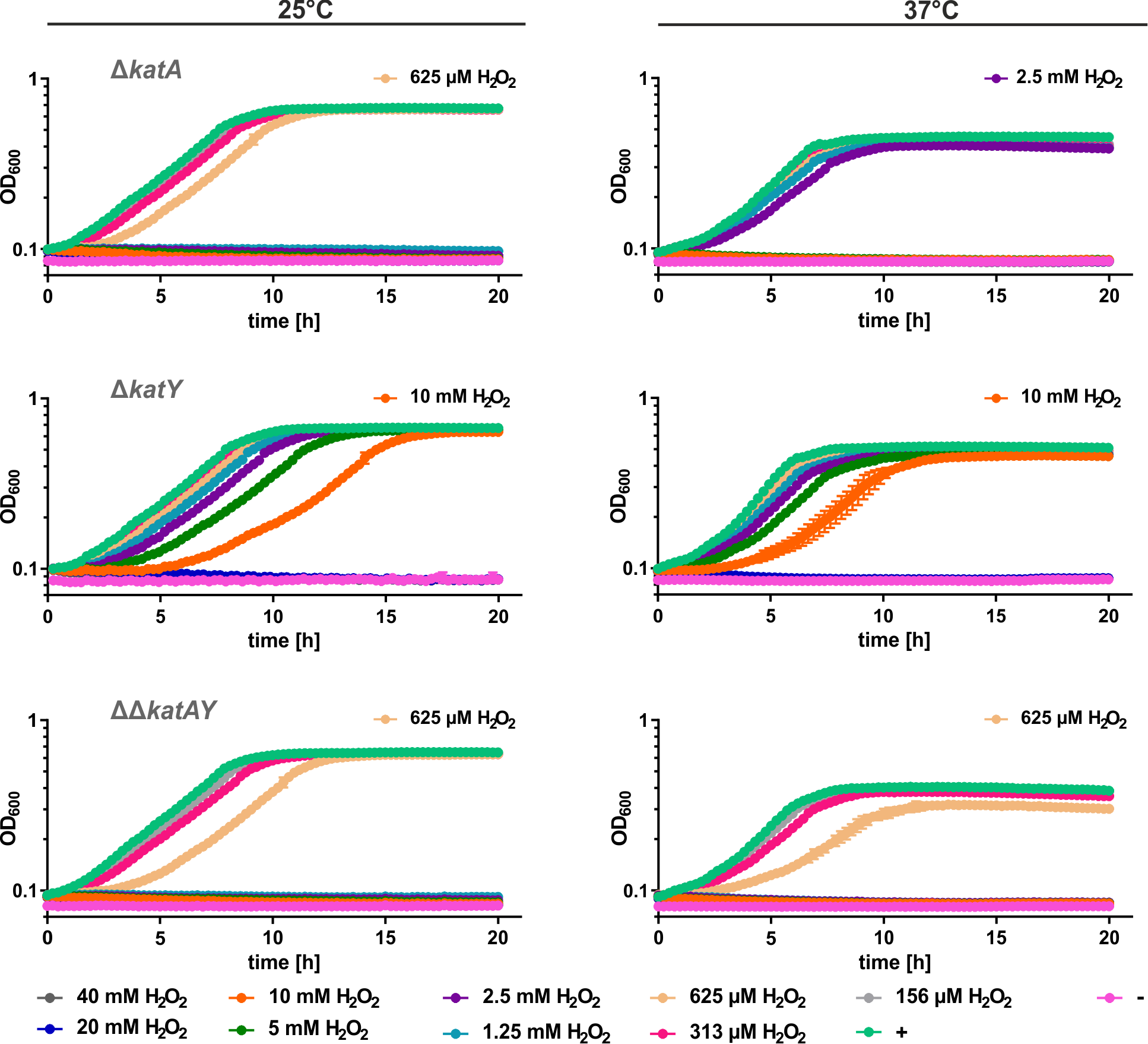

Supplement: S2 Fig — (TIF) [file pgen.1010669.s002.tif]

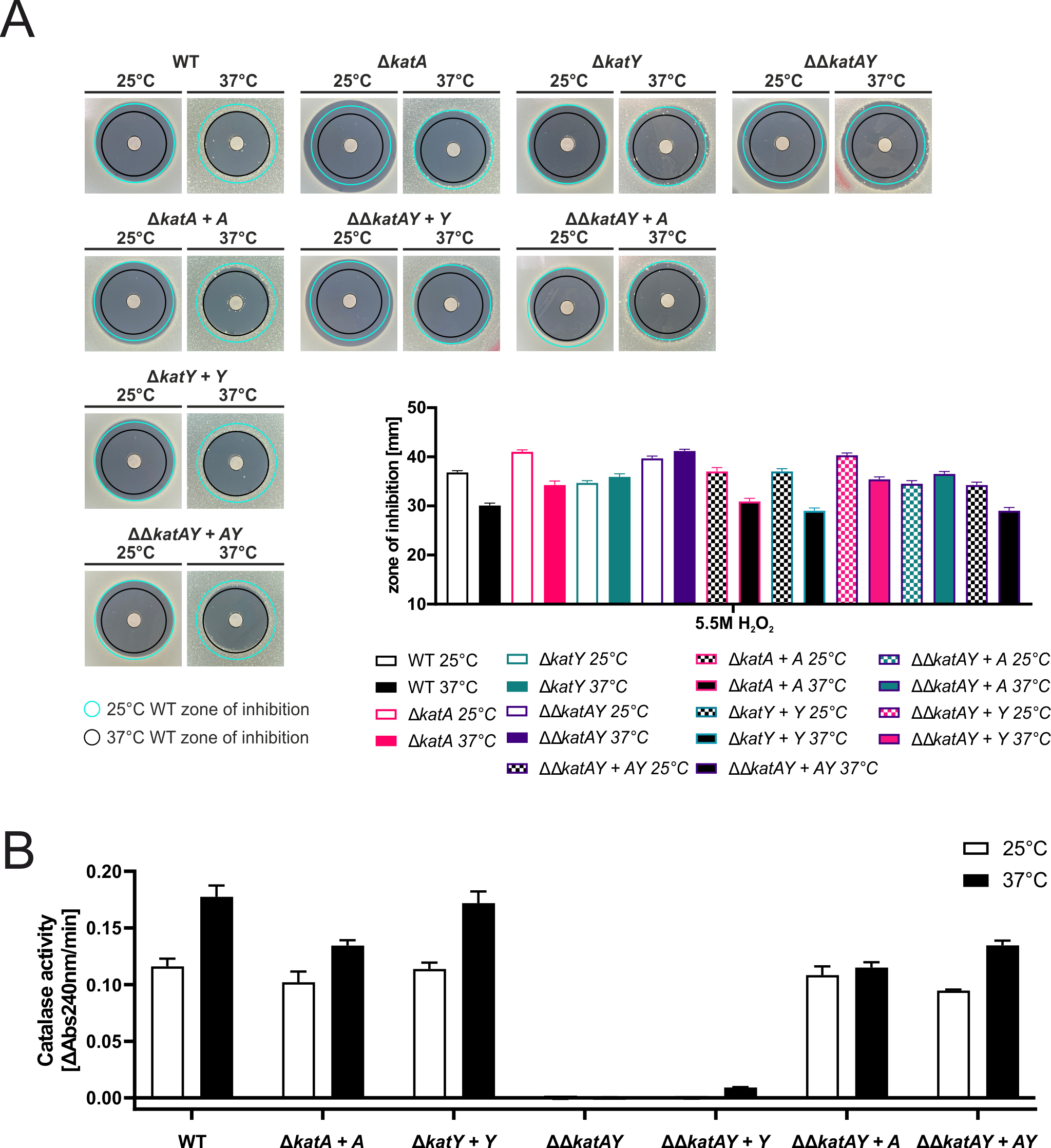

Supplement: S3 Fig — (TIF) [file pgen.1010669.s003.tif]

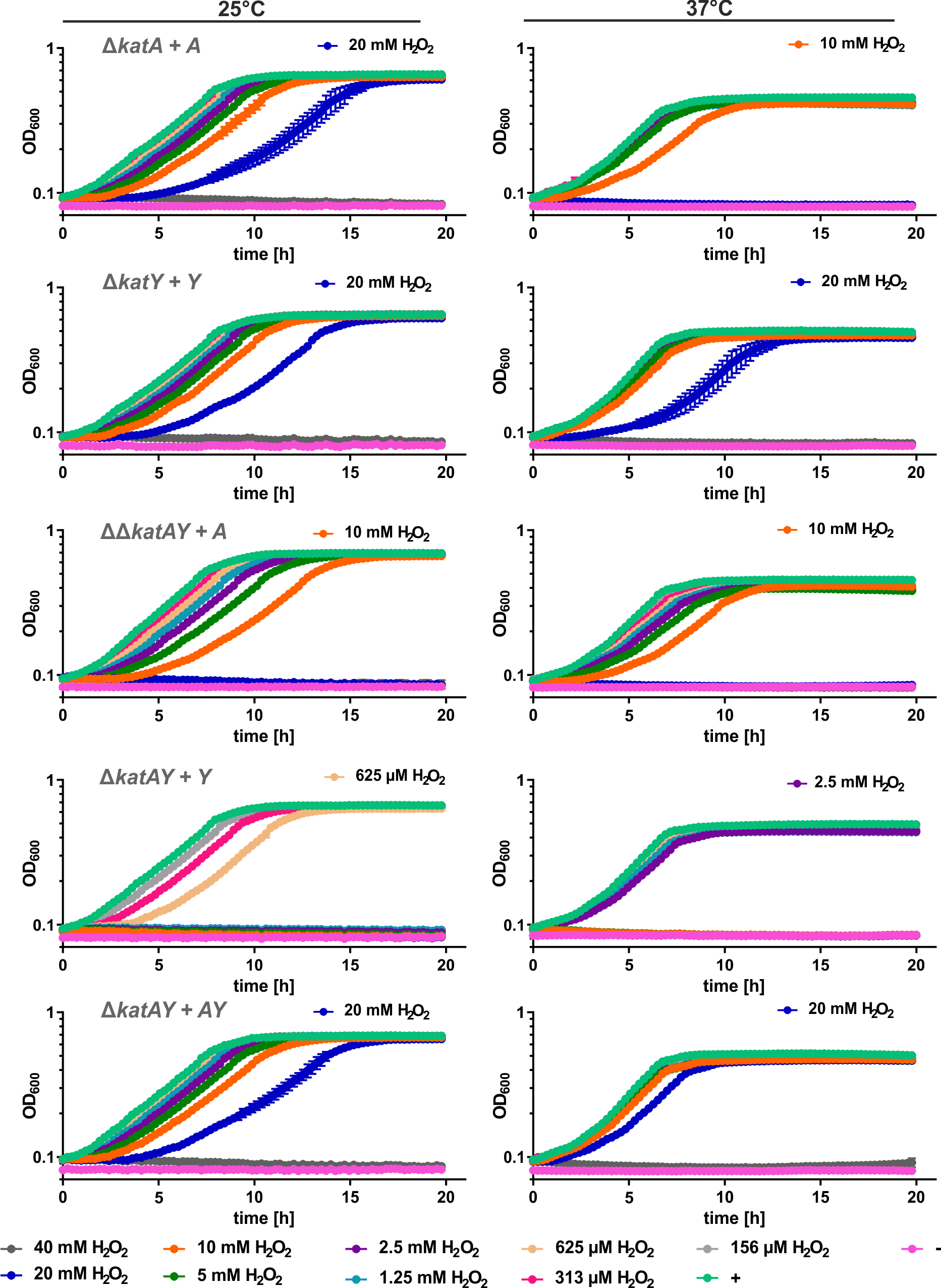

Supplement: S4 Fig — (TIF) [file pgen.1010669.s004.tif]

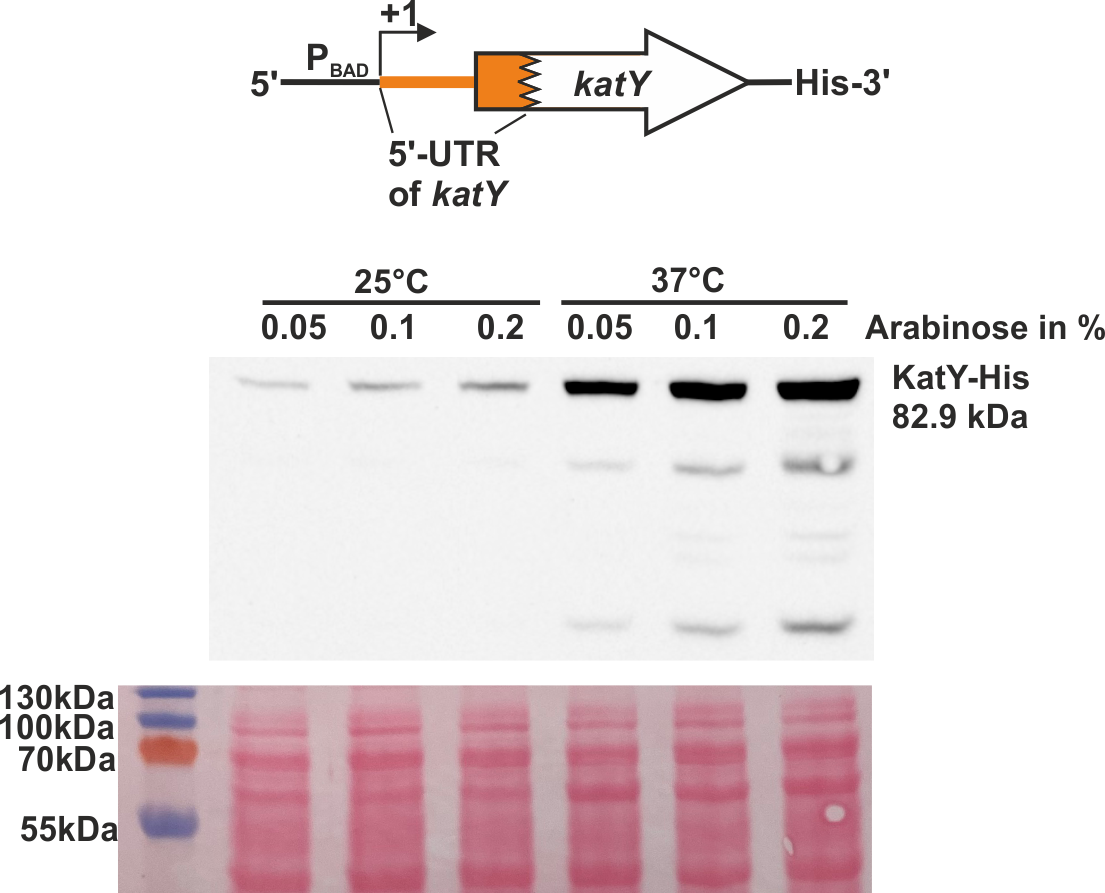

Supplement: S5 Fig — (TIF) [file pgen.1010669.s005.tif]

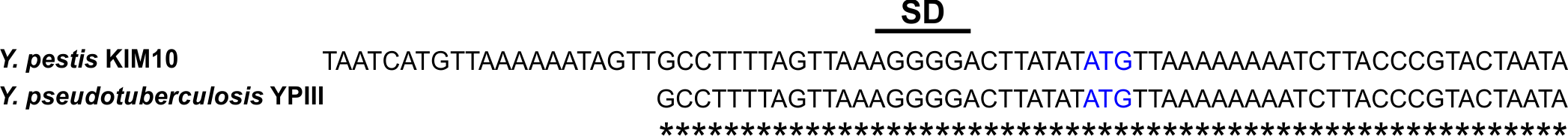

Supplement: S6 Fig — (TIF) [file pgen.1010669.s006.tif]
