# Supplementary material for: The oxidative stress response, in particular the katY gene, is temperature-regulated in Yersinia pseudotuberculosis
Source: PLoS Genet. 2023 Jul 10;19(7):e1010669. doi: 10.1371/journal.pgen.1010669 (PMC10358904; doi:10.1371/journal.pgen.1010669)
Supplement: S1 Table — (DOCX) [file pgen.1010669.s007.docx]

**Table S1: Bacterial strains**

| **Strain** | **Relevant characteristics** | **Reference** |
| --- | --- | --- |
| ***Yersinia pseudotuberculosis*** |  |  |
| YPIII | pIB1, wild type | [1] |
| YPIII Δ*katA* | pIB1 Δ*katA* | This study |
| YPIII Δ*katY* | pIB1 Δ*katY* | This study |
| YPIII ΔΔ*katAY* | pIB1 ΔΔ*katAY* | This study |
| YPIII Δ*katA + katA-*His | pIB1 Δ*katA;* reintroduction of His-tagged KatA by homologous recombination | This study |
| YPIII Δ*katY + katY-*His | pIB1 Δ*katY;* reintroduction of His-tagged KatY by homologous recombination | This study |
| YPIII ΔΔ*katAY + katA-*His | pIB1 ΔΔ*katAY;* reintroduction of His-tagged KatA by homologous recombination | This study |
| YPIII ΔΔ*katAY + katY-*His | pIB1 ΔΔ*katAY;* reintroduction of His-tagged KatY by homologous recombination | This study |
| YPIII ΔΔ*katAY + katA-*His and *katY-*His | pIB1 ΔΔ*katAY + katA*-His*;* reintroduction of His-tagged KatY by homologous recombination | This study |
| ***Escherichia coli*** |  |  |
| DH5α | *supE44*, Δ*lacU169* (ψ80*lacZ*Δ*M15*), *hsdR17*, *recA1*, *gyrA96*, *thi1*, *relA1* | [2] |
| S17-1 *λpir* | *RP4-2 Tc::Mu-Km::Tn7 (λpir)* | [3] |

**References**

1. Bolin I, Norlander L, Wolf-Watz H. Temperature-inducible outer membrane protein of *Yersinia pseudotuberculosis* and *Yersinia enterocolitica* is associated with the virulence plasmid. Infection and Immunity. 1982;37: 506–512.

2. Hanahan D. Studies on transformation of *Escherichia coli* with plasmids. Journal of Molecular Biology. 1983;166: 557–580. doi:10.1016/S0022-2836(83)80284-8

3. Simon R, Priefer U, Pühler A. A broad host range mobilization system for *in vivo* genetic engineering: Transposon mutagenesis in Gram negative bacteria. Nat Biotechnol. 1983;1: 784–791. doi:10.1038/nbt1183-784
