# Supplementary material for: The oxidative stress response, in particular the katY gene, is temperature-regulated in Yersinia pseudotuberculosis
Source: PLoS Genet. 2023 Jul 10;19(7):e1010669. doi: 10.1371/journal.pgen.1010669 (PMC10358904; doi:10.1371/journal.pgen.1010669)
Supplement: S2 Table — (DOCX) [file pgen.1010669.s008.docx]

**Table S2: Plasmid list**

| **Plasmid** | **Relevant characteristics** | **Reference** |
| --- | --- | --- |
| pUC18 | Cloning vector; Ap^r^ | [1] |
| pDM4 | *sacBR, oriT, oriR6K,* Cm*^r^* | [2] |
| pCC_roGFP2-orp1 | Redox sensitive probe*, roGFP2-orp1; ptac* | [3] |
| pBAD2-*bgaB*-His | *bgaB* reporter gene vector, Ap^r^, araC, P_BAD_ promoter, His‑Tag at the C‑terminal end of BgaB | [4] |
| pBAD-His A | Expression vector, Ap^r^, araC, P_BAD_ promoter, N-terminal His‑Tag | Invitrogen, Carlsbad, CA |
| pBO6202 | pBAD2-bgaB-His, short 5’-UTR of *yopN* (pYP0065) plus 30 bp of the coding region, (-37 to +30 bp from *yopN* ATG) | [5] |
| pBO4423 | pBAD2-bgaB-His; 5’-UTR of *sodA* (YPK_0035) plus 30 bp of *sodA* coding region (-55 to +30 bp from *sodA* ATG) | [4] |
| pBO4406 | pBAD2-bgaB-His; 5’-UTR of *sodB* (YPK_1863) plus 30 bp of *sodB* coding region (-89 to +30 bp from *sodB* ATG) | [4] |
| pBO4902 | pBAD2-bgaB-His; 5’-UTR of *sodB* (YPK_1863) plus 30 bp of *sodB* coding region (-89 to +30 bp from *sodB* ATG), mutant Rep AA70-71CT,C73T | This study |
| pBO4914 | pUC18; YPK_1863 (*sodB*) 5’-UTR plus coding region (-89 to +60 bp from *sodB* ATG); runoff plasmid for structure probing and primer extension inhibition | This study |
| pBO4917 | pUC18; YPK_1863 (*sodB*) 5’-UTR plus coding region (-89 to +60 bp from *sodB* ATG), mutant Rep AA70-71CT,C73T; runoff plasmid for structure probing and primer extension inhibition | This study |
| pBO4909 | pBAD2-bgaB-His; 5’-UTR of *sodC* (YPK_3445) plus 30 bp of *sodC* coding region (-63 to +30 bp from *sodC* ATG) | This study |
| pBO6861 | pBAD2-bgaB-His; 5’-UTR of *sodC* (YPK_3445) plus 30 bp of *sodC* coding region (-63 to +30 bp from *sodC* ATG) mutant Rep TG83-84CC | This study |
| pBO4916 | pUC18; YPK_3445 (*sodC*) 5’-UTR plus coding region (-63 to +60 bp from *sodC* ATG); runoff plasmid for structure probing and primer extension inhibition | This study |
| pBO6864 | pUC18; YPK_3445 (*sodC*) 5’-UTR plus coding region (-63 to +60 bp from *sodC* ATG), mutant Rep TG83-84CC; runoff plasmid for structure probing and primer extension inhibition | This study |
| pBO4416 | pBAD2-bgaB-His; 5’-UTR of *katA* (YPK_2855) plus 30 bp of *katA* coding region (-121 to +30 bp from katA ATG) | [4] |
| pBO4905 | pBAD2-bgaB-His; 5’-UTR of *katA* (YPK_2855) plus 30 bp of *katA* coding region (-121 to +30 bp from katA ATG) Mutant Rep AC109-110CG | This study |
| pBO4915 | pUC18; YPK_2855 (*katA*) 5’-UTR plus coding region (-121 to +60 bp from *katA* ATG); runoff plasmid for structure probing and primer extension inhibition | This study |
| pBO4918 | pUC18; YPK_2855 (*katA*) 5’-UTR plus coding region (-121 to +60 bp from *katA* ATG), mutant Rep AC109-110CG; runoff plasmid for structure probing and primer extension inhibition | This study |
| pBO4436 | pBAD2-bgaB-His; 5’-UTR of *katY* (YPK_3388) plus 30 bp of *katY* coding region (-26 to +30 bp from *katY* ATG) | This study |
| pBO6887 | pBAD2-bgaB-His; 5’-UTR of *katY* (YPK_3388) plus 30 bp of *katY* coding region (-26 to +30 bp from *katY* ATG) mutant Rep T31C | This study |
| pBO6886 | pBAD2-bgaB-His; 5’-UTR of *katY* (YPK_3388) plus 30 bp of *katY* coding region (-26 to +30 bp from *katY* ATG) mutant Derep TA31-32CT | This study |
| pBO7248 | pUC18; YPK_3388 (*katY*) 5’-UTR plus coding region (-26 to +30 bp from *katA* ATG); runoff plasmid for structure probing | This study |
| pBO7238 | pUC18; YPK_3388 (*katY*) 5’-UTR plus coding region (-26 to +60 bp from *katA* ATG); runoff plasmid for primer extension inhibition | This study |
| pBO3179 | pBAD2-bgaB-His; short 5’-UTR of *trxA* (YPK_4035) plus 30 bp of *trxA* coding region (-58 to +30 bp from *trxA* ATG) | [4] |
| pBO6859 | pBAD2-bgaB-His; short 5’-UTR of *trxA* (YPK_4035) plus 30 bp of *trxA* coding region (-58 to +30 bp from *trxA* ATG) Mutant Rep A24T | This study |
| pBO3178 | pBAD2-bgaB-His; long 5’-UTR of *trxA* (YPK_4035) plus 30 bp of *trxA* coding region (-98 to +30 bp from *trxA* ATG) | [4] |
| pBO6862 | pBAD2-bgaB-His; long 5’-UTR of *trxA* (YPK_4035) plus 30 bp of *trxA* coding region (-98 to +30 bp from *trxA* ATG) Mutant Rep A79C | This study |
| pBO6857 | pUC18; short YPK_4035 (*trxA*) 5’-UTR plus coding region (-58 to +60 bp from *trxA* ATG); runoff plasmid for structure probing and primer extension inhibition | This study |
| pBO6858 | pUC18; short YPK_4035 (*trxA*) 5’-UTR plus coding region (-58 to +60 bp from *trxA* ATG), mutant Rep A24T; runoff plasmid for structure probing and primer extension inhibition | This study |
| pBO6863 | pUC18; long YPK_4035 (*trxA*) 5’-UTR plus coding region (-98 to +60 bp from *trxA* ATG); runoff plasmid for structure probing and primer extension inhibition | This study |
| pBO7246 | pBAD-His A Expression vector, 5’-UTR and *katY* (YPK_3388) with a His-tag at the C-terminal end | This study |
| pBO6868 | pDM4, *katA* deletion fragment for generation of Δ*katA* by bacterial conjugation | This study |
| pBO7212 | pDM4, *katY* deletion fragment for generation of Δ*katY* by bacterial conjugation | This study |
| pBO6888 | pDM4, *katA*-His complementation fragment for restoration of *katA* with a C-terminal His-tag by bacterial conjugation | This study |
| pBO7251 | pDM4, *katY*-His complementation fragment for restoration of *katY* with a C-terminal His-tag by bacterial conjugation | This study |

**References**

1. Yanisch-Perron C, Vieira J, Messing J. Improved Ml3 phage cloning vectors and host strains: Nucleotide sequences of the M13mp18 and pUC19 vectors. Gene. 1985;33: 103–119. doi:10.1016/0378-1119(85)90120-9

2. Milton DL, O’Toole R, Horstedt P, Wolf-Watz H. Flagellin A is essential for the virulence of *Vibrio anguillarum*. J Bacteriol. 1996;178: 1310–1319. doi:10.1128/jb.178.5.1310-1319.1996

3. Degrossoli A, Müller A, Xie K, Schneider JF, Bader V, Winklhofer KF, et al. Neutrophil-generated HOCl leads to non-specific thiol oxidation in phagocytized bacteria. Winterbourn C, editor. eLife. 2018;7: e32288. doi:10.7554/eLife.32288

4. Righetti F, Nuss AM, Twittenhoff C, Beele S, Urban K, Will S, et al. Temperature-responsive *in vitro* RNA structurome of *Yersinia pseudotuberculosis*. Proceedings of the National Academy of Sciences. 2016;113: 7237–7242. doi:10.1073/pnas.1523004113

5. Pienkoß S, Javadi S, Chaoprasid P, Nolte T, Twittenhoff C, Dersch P, et al. The gatekeeper of *Yersinia* type III secretion is under RNA thermometer control. PLOS Pathogens. 2021;17: e1009650. doi:10.1371/journal.ppat.1009650
