# Supplementary material for: The oxidative stress response, in particular the katY gene, is temperature-regulated in Yersinia pseudotuberculosis
Source: PLoS Genet. 2023 Jul 10;19(7):e1010669. doi: 10.1371/journal.pgen.1010669 (PMC10358904; doi:10.1371/journal.pgen.1010669)
Supplement: S3 Table — (DOCX) [file pgen.1010669.s009.docx]

**Table S3: Oligonucleotide list**

| **Name** | **Purpose** | **Plasmid** | **Sequence (5‘->3‘)** |
| --- | --- | --- | --- |
| sodB_ro_fw | forward primer to amplify YPK_1863 (*sodB*) 5’-UTR with a T7 promoter for the construction of the runoff plasmid | pBO4914 | AGAAATTAATACGACTCACTATAGGGTTTTAACCGCTCTTCACCC |
| sodB_ro_rev | reverse primer to amplify YPK_1863 (*sodB*) 5’-UTR + 60 bp from ATG; reverse primer with Nael site for the construction of the runoff plasmid for structure probing and primer extension inhibition | pBO4914 | GCCGGCAGAGATGTGGGGTTCCAG |
| sodB_rep_fw | mutagenesis forward primer to introduce the mutation AA70-71CT, C73T into YPK_1863 (*sodB*) 5‘-UTR | pBO4902 pBO4917 | GAGCAAACACCCTGAGCCTCTCCGAAAGGAGAGAGCT |
| sodB_rep_rev | mutagenesis reverse primer to introduce the mutation AA70-71CT, C73T into YPK_1863 (*sodB*) 5‘-UTR | pBO4902 pBO4917 | AGCTCTCTCCTTTCGGAGAGGCTCAGGGTGTTTGCTC |
| sodC_UTR_fw | forward primer to amplify the 5’-UTR of YPK_3445 (*sodC*) plus 30 bp of *sodC* coding region (-63 to +30 bp from *sodC* ATG) | pBO4909 | TTGCTAGCGTCAAAAGTTTCCTATTGACAA |
| sodC_UTR_rev | reverse primer to amplify the 5’-UTR of YPK_3445 (*sodC*) plus 30 bp of *sodC* coding region (-63 to +30 bp from *sodC* | pBO4909 | TTGAATTCAACAGGTAGCAATAATGTAATT |
| sodC_ro_fw | forward primer to amplify YPK_3445 (*sodC*) 5’-UTR with a T7 promoter for the construction of the runoff plasmid | pBO4916 | AGAAATTAATACGACTCACTATAGGGGTCAAAAGTTTCCTATTGACAA |
| sodC_ro_rev | reverse primer to amplify YPK_3445 (*sodC*) 5’-UTR + 60 bp from ATG; reverse primer with Nael site for the construction of the runoff plasmid for structure probing and primer extension inhibition | pBO4916 | GCCGGCAGCGGCCAGTGTTGCG |
| sodC_rep_fw | mutagenesis forward primer to introduce the mutation TG83-84CC into YPK_3445 (*sodC*) 5‘-UTR | pBO6861 pBO6864 | TGAAATTAATTACATTATCCCTACCTGTTATTCTTTAC |
| sodC_rep_rev | mutagenesis reverse primer to introduce the mutation TG83-84CC into YPK_3445 (*sodC*) 5‘-UTR | pBO6861 pBO6864 | GTAAAGAATAACAGGTAGGGATAATGTAATTAATTTCA |
| katA_ro_fw | forward primer to amplify YPK_2855 (*katA*) 5’-UTR with a T7 promoter for the construction of the runoff plasmid | pBO4915 | AGAAATTAATACGACTCACTATAGGGGAGATCAAAGAATAACACCGT |
| katA_ro_rev | reverse primer to amplify YPK_2855 (*katA*) 5’-UTR + 60 bp from ATG; reverse primer with Nael site for the construction of the runoff plasmid for structure probing and primer extension inhibition | pBO4915 | GCCGGCATTATTATTATCGACAACGGG |
| katA_rep_fw | mutagenesis forward primer to introduce the mutation AC109-110CG into YPK_2855 (*katA*) 5‘-UTR | pBO4905 pBO4918 | CAATCAATTCAACAGCCATCGTGGAGAACATGATGAG |
| katA_rep_rev | mutagenesis reverse primer to introduce the mutation AC109-110CG into YPK_2855 (*katA)* 5‘-UTR | pBO4905 pBO4918 | CTCATCATGTTCTCCACGATGGCTGTTGAATTGATTG |
| katA_5‘-flank_fw | forward primer to amplify the upstream region (683 bp) of YPK_2855 (*katA)* for deletion of *katA* | pBO6868 | TTTGTCGACTTGATCGGATAGCTTAGCCC |
| katA_5‘-flank rev | reverse primer to amplify the upstream region (683 bp) of YPK_2855 (*katA)* with an overlap to the downstream region (403 bp) of *katA* for deletion of *katA* | pBO6868 | TTCAAAGGGGCTGATCGGGTCATGTTCTCCAGTATGGCTG |
| katA_3’flank_fw | forward primer to amplify the downstream region (403 bp) of YPK_2855 (*katA)* with an overlap to the upstream region (683 bp) for deletion of *katA* | pBO6868 | CAGCCATACTGGAGAACATGACCCGATCAGCCCCTTTGAA |
| katA_3’flank_rev | reverse primer to amplify the upstream region (403 bp) of YPK_2855 (*katA)* for deletion of *katA* | pBO6868 | TTTTCTAGATCACTGAGGGAGAGTGGTTA |
| katA_EP_fw | forward primer to check for deletion/complementation of YPK_2855 (*katA)* | - | AATACGTTCCAACGCACCCC |
| katA_EP_rev | reverse primer to check for deletion/complementation of YPK_2855 (*katA)* | - | TTATCGGCTAGTCGAAGAGC |
| katA_IP_fw | forward primer to check for deletion/complementation of YPK_2855 (*katA)* | - | ACGTGATCCACTGAAGTTCC |
| katA_IP_rev | reverse primer to check for deletion/complementation of YPK_2855 (*katA)* | - | CTTACCGTCATATGCACGG |
| katA-His_fw | forward primer to amplify the 5’-UTR of YPK_2855 (*katA)* plus *katA* with a His-tag for complementation of Δ*katA* | pBO6888 | TTTCCATGGGAGATCAAAGAATAACACCGTG |
| katA-His_rev | reverse primer to amplify the 5’-UTR of YPK_2855 (*katA)* plus *katA* with a His-tag for complementation of Δ*katA* | pBO6888 | TTTTTAGTGGTGATGGTGATGATGATTCAGGCCAAGTGCTTTTTTCA |
| pDM4_katA_Del_fw | forward primer to linearize the pDM4-*katA*-deletion plasmid for NEB Hifi Assembly | pBO6888 | CATGTTCTCCAGTATGGCTG |
| pDM4_katA_Del_rev | reverse primer to linearize the pDM4-*katA*-deletion plasmid for NEB Hifi Assembly | pBO6888 | ACCCGATCAGCCCCTTTG |
| katA_pDM4_Del_fw | forward primer to amplify the 5’-UTR of YPK_2855 (*katA)* plus *katA* with a His-tag and an overlap to the pDM4-*katA*-deletion plasmid for complementation of Δ*katA* | pBO6888 | TTCAAAGGGGCTGATCGGGTTTAGTGGTGATGGTGATG |
| katA_pDM4_Del_rev | reverse primer to amplify the 5’-UTR of YPK_2855 (*katA)* plus *katA* with a His-tag and an overlap to the pDM4-*katA*-deletion plasmid for complementation of Δ*katA* | pBO6888 | CAGCCATACTGGAGAACATGATGAGCAAGAAGAAAGGATTAAC |
| katY_UTR_fw | forward primer to amplify the 5’-UTR of YPK_3388 (*katY*) plus 30 bp of *katY* coding region (-26 to +30 bp from *katY* ATG) | pBO4436 | TTGCTAGCGCCTTTTAGTTAAAGGGGAC |
| katY_UTR_rev | reverse primer to amplify the 5’-UTR of YPK_3388 (*katY*) plus 30 bp of *katY* coding region (-26 to +30 bp from *katY* ATG | pBO4436 | TTGAATTCTATTAGTACGGGTAAGATTTTTTT |
| katY_ro_fw | forward primer to amplify YPK_3388 (*katY*) 5’-UTR with a T7 promoter for the construction of the runoff plasmids | pBO7238 pBO7248 | AGAAATTAATACGACTCACTATAGGGGCTAGCGCCTTTTAGTTAAAGG |
| katY_ro_30nt_rev | reverse primer to amplify YPK_3388 (*katY*) 5’-UTR + 30 bp from ATG; reverse primer with EcoRV site for the construction of the runoff plasmid for structure probing and primer extension inhibition | pBO7248 | AAGATATCTATTAGTACGGGTAAGATTTTTT |
| katY_ro_rev | reverse primer to amplify YPK_3388 (*katY*) 5’-UTR + 60 bp from ATG; reverse primer with EcoRV site for the construction of the runoff plasmid for structure probing and primer extension inhibition | pBO7238 | AAGATATCCGTAGGTGTATTATGTACAATGGC |
| katY_rep_fw | mutagenesis forward primer to introduce the mutation T31C into YPK_3388 (*katY*) 5‘-UTR | pBO6887 | AAGGGGACTTATATATGTCAAAAAAAATCTTACCCGT |
| katY_rep_rev | mutagenesis reverse primer to introduce the mutation T31C into YPK_3388 (*katY*) 5‘-UTR | pBO6887 | ACGGGTAAGATTTTTTTTGACATATATAAGTCCCCTT |
| katY_derep_fw | mutagenesis forward primer to introduce the mutation TA31-32CT into YPK_3388 (*katY*) 5‘-UTR | pBO6886 | AAGGGGACTTATATATGTCTAAAAAAATCTTACCCGTA |
| katY_derep_rev | mutagenesis reverse primer to introduce the mutation TA31-32CT into YPK_3388 (*katY*) 5‘-UTR | pBO6886 | TACGGGTAAGATTTTTTTAGACATATATAAGTCCCCTT |
| katY_5‘-flank_fw | forward primer to amplify the upstream region (468 bp) of YPK_3388 (*katY)* for deletion of *katY* | pBO7212 | TTTGTCGACTACGCCTTGTCCAATGTCAG |
| katY_5‘-flank rev | reverse primer to amplify the upstream region (468 bp) of YPK_3388 (*katY)* with an overlap to the downstream region (603 bp) of *katY* for deletion of *katY* | pBO7212 | ATCCTCCCCTCTATTTAGATATAAGTCCCCTTTAAC |
| katY_3’flank_fw | forward primer to amplify the downstream region (603 bp) of YPK_3388 (*katY)* with an overlap to the upstream region (468 bp) for deletion of *katY* | pBO7212 | GTTAAAGGGGACTTATATCTAAATAGAGGGGAGGAT |
| katY_3’flank_rev | reverse primer to amplify the upstream region (603 bp) of YPK_3388 (*katY)* for deletion of *katY* | pBO7212 | TTTTCTAGATTCCTGAAATATACCCGTCGC |
| katY_EP_fw | forward primer to check for deletion/complementation of YPK_3388 (*katY)* | - | CGGTAAGACAGGATTTCAGTAGGG |
| katY_EP_rev | reverse primer to check for deletion/complementation of YPK_3388 (*katY)* | - | GACTAGGCTTCGGTACTATTGGTG |
| katY_IP_fw | forward primer to check for deletion/complementation of YPK_3388 (*katY)* | - | CCTTACAGGCAATATCGCGC |
| katY_IP_rev | reverse primer to check for deletion/complementation of YPK_3388 (*katY)* | - | GGGTTGATTTACGTAAACCCT |
| pBAD-His_fw | forward primer to linearize the pBAD-His A plasmid for NEB Hifi Assembly | pBO7246 | GGTACCATATGGGAATTCGAAG |
| pBAD-His_rev | reverse primer to linearize the pBAD-His A plasmid for NEB Hifi Assembly | pBO7246 | GGTATGGAGAAACAGTAGAG |
| katY-His_fw | forward primer to amplify the 5’-UTR of YPK_3388 (*katY)* plus *katY* with a His-tag for complementation of Δ*katY* | pBO7246 pBO7251 | TTTCCATGGGCCTTTTAGTTAAAGGGGACTT |
| katY-His_rev | reverse primer to amplify the 5’-UTR of YPK_3388 (*katY)* plus *katY* with a His-tag for complementation of Δ*katY* | pBO7246 pBO7251 | TTTTTAGTGGTGATGGTGATGATGGTTATTTTTTATATCAAAGCGATCA |
| katY_pBAD_fw | forward primer to amplify the 5’-UTR of YPK_3388 (*katY)* plus *katY* with a His-tag and an overlap to the pBAD-His plasmid for complementation of Δ*katY* | pBO7246 | CTCTACTGTTTCTCCATACCGCCTTTTAGTTAAAGGGGAC |
| His__pBAD_rev | reverse primer to amplify the 5’-UTR of YPK_3388 (*katY)* plus *katY* with a His-tag and an overlap to the pBAD-His plasmid for complementation of Δ*katY* | pBO7246 | TCGAATTCCCATATGGTACCTTAGTGGTGATGGTGATGATG |
| pDM4_katY_Del_fw | forward primer to linearize the pDM4- *katY* -deletion plasmid for NEB Hifi Assembly | pBO7251 | ATATAAGTCCCCTTTAACTAAAAGGC |
| pDM4_katY_Del_rev | reverse primer to linearize the pDM4-*katA*-deletion plasmid for NEB Hifi Assembly | pBO7251 | CTAAATAGAGGGGAGGATTTATC |
| katY_pDM4_Del_fw | forward primer to amplify the 5’-UTR of YPK_3388 (*katY)* plus *katY* with a His-tag and an overlap to the pDM4- *katY* -deletion plasmid for complementation of Δ*katY* | pBO7251 | AAATCCTCCCCTCTATTTAGTTAGTGGTGATGGTGATG |
| katY_pDM4_Del_rev | reverse primer to amplify the 5’-UTR of YPK_3388 (*katY)* plus *katY* with a His-tag and an overlap to the pDM4-*katY*-deletion plasmid for complementation of Δ*katY* | pBO7251 | TAGTTAAAGGGGACTTATATATGTTAAAAAAAATCTTACCCG |
| trxAshort_ro_fw | forward primer to amplify YPK_4035 (*trxA*) short 5’-UTR with a T7 promoter for the construction of the runoff plasmid | pBO6857 | AGAAATTAATACGACTCACTATAGGGGAGATCAAAGAATAACACCGT |
| trxAshort_ro_rev | reverse primer to amplify YPK_4035 (*trxA*) short 5’-UTR + 60 bp from ATG; reverse primer with EcoRV site for the construction of the runoff plasmid for structure probing and primer extension inhibition | pBO6857 | GCCGGCATTATTATTATCGACAACGGG |
| trxAshort_rep_fw | mutagenesis forward primer to introduce the mutation A24T into YPK_4035 (*trxA*) 5‘-UTR | pBO6858 pBO6859 | CTACTGTTGGTTAATGCTACTCCAACGAGGTAGACACAATC |
| trxAshort_rep_rev | mutagenesis reverse primer to introduce the mutation A24T into YPK_4035 (*trxA*) 5‘-UTR | pBO6858 pBO6859 | GATTGTGTCTACCTCGTTGGAGTAGCATTAACCAACAGTAG |
| trxAlong_rep_fw | mutagenesis forward primer to introduce the mutation A24T into YPK_4035 (*trxA*) 5‘-UTR | pBO6862 | GCTACACCAACGAGGTAGACCCAATCCTTTGGAGTAGAACA |
| trxAlong_rep_rev | mutagenesis reverse primer to introduce the mutation A24T into YPK_4035 (*trxA*) 5‘-UTR | pBO6862 | TGTTCTACTCCAAAGGATTGGGTCTACCTCGTTGGTGTAGC |
| RT_sodB_fw | forward primer for detection of YPK_1863 (*sodB)* by qRT-PCR | - | CTGCCGAAACGCTGGAATACCATTATGG |
| RT_sodB_rev | reverse primer for detection of YPK_1863 (*sodB)* by qRT-PCR | - | GATCTCTTCCAGTGATTTGCCTGC |
| RT_sodC_fw | forward primer for detection of YPK_3445 (*sodC)* by qRT-PCR | - | GGGATTGGTGGTTAATGCAGATGG |
| RT_sodC_rev | reverse primer for detection of YPK_3445 (*sodC)* by qRT-PCR | - | CACCGCCAGCATGGATCATTAAC |
| RT_katA_fw | forward primer for detection of YPK_2855 (*katA)* by qRT-PCR | - | GACACCGACTATTTCTCTCAACCACG |
| RT_katA_rev | reverse primer for detection of YPK_2855 (*katA)* by qRT-PCR | - | GCTTCAGGAACTTGCGATAACTCACC |
| RT_katY_fw | forward primer for detection of YPK_3388 (*katY)* by qRT-PCR | - | GGGCGAATAAACTGGAACTGACC |
| RT_katY_rev | reverse primer for detection of YPK_3388 (*katY)* by qRT-PCR | - | CGTATTCGTCAACACACCAGCCTTAG |
| RT_bgaB_fw | forward primer for detection of *bgaB* by qRT-PCR | - | GACTGCAACTACTCCAGCTTGGTTTG |
| RT_bgaB_rev | reverse primer for detection of *bgaB* by qRT-PCR | - | CTACTGCCAAACGAGAGAATGACACC |
| RT_nuoB_fw | forward primer for detection of YPK_1561 (*nuoB)* by qRT-PCR | - | GATCCTCTCGAGCAACATG |
| RT_nuoB_rev | reverse primer for detection of YPK_1561 (*nuoB)* by qRT-PCR | - | TAAAGCAGGTTCCGGCCA |
| RT_gyrB_fw | forward primer for detection of YPK_0004 (*gyrB)* by qRT-PCR | - | TCGCCGTGAAGGTAAAGTTC |
| RT_gyrB_rev | reverse primer for detection of YPK_0004 (*gyrB)* by qRT-PCR | - | CGTAATGGAAGTGGTCTTCT |
