# Supplementary material for: The oxidative stress response, in particular the katY gene, is temperature-regulated in Yersinia pseudotuberculosis
Source: PLoS Genet. 2023 Jul 10;19(7):e1010669. doi: 10.1371/journal.pgen.1010669 (PMC10358904; doi:10.1371/journal.pgen.1010669)
Supplement: Attachment — Submitted filename: Scheller_rebuttal.pdf [file pgen.1010669.s015.pdf]

Dear Jade and Lotte,

Thank you very much for giving us the opportunity to submit a revised version of manuscript PGENETICS-D-23-00174 by Daniel Scheller and co-authors. We appreciate the overall positive and constructive comments from the reviewers.

As you will see below, we carefully considered all reviewer comments and revised the manuscript accordingly. We feel that the manuscript has substantially improved.

Changes in the revised version are highlighted in yellow in the marked manuscript. We are confident that you will now find our comprehensive study acceptable for PLoS Genetics.

Thank you very much for handling our manuscript.

Franz

Our point-by-point response to the reviewers is as follows:

Reviewer #1:
This study investigates … …
The manuscript is both clear and well written. The description of the temperature-dependent mechanism of mRNA translation activation followed by functional assays of H2O2 scavenging is quite convincing. I have a few specific comments in order to improve the quality of the manuscript.

Thank you! We very much appreciate this positive judgement.

Major comments:
1. Results in Figure 2 is missing some important points. How long was the incubation when cells were incubated from 25'C to 37'C? Please add a positive control (e.g yopN) in the figure.

As suggested, *yopN* was added as a positive control, which shows essentially no transcriptional control but is regulated by an efficient RNAT (Pienkoß et al., PLoS Pathogens, 2021). Moreover, the missing information was added to the figure legend.

2. Figure 3. Is the Western blot for Y pseudotuberculosis on the left reversed? There is a discrepancy between the B-galactosidase results and the Western blot signals (although the Western blot is not clearly indicated on the figure). Is this difference due to the decrease in transcript observed in Fig 3D? The only result fitting the Western blot and B-gal assays is the control yopN. All the others, Soda, SodB and SodC, are way stronger when extracted from 25'C compared to 37'C.

No, the Western blot is not reversed. We repeated the experiment multiple times because we were puzzled by the apparent discrepancy in the *Yersinia* results. This may have several reasons, most apparently the reduced transcript levels shown in Fig. 3D (see revised Results and Discussion). These results actually were the reason why we focused our attention on the katalase genes, in particular the newly discovered *katY* RNAT.

Please note that not only the control *yopN* showed a "fitting result". This was also true for *trxA*(s) and even more for *katY*, which was the major incentive for the present study.

3. Figure 4. It would be interesting to visualize the WT and mutated (rep A24U) trxA mRNA by Northern or qRT-PCR. Although the results suggest a strong translational repression of trxA A24U, it is possible that the transcript becomes highly unstable due to the mutation. Please discuss this.

Yes, in principle "it is possible" that the mutated transcript becomes unstable due to the mutation, and it "would be interesting to visualize" the mRNAs. The manuscript already presents an enormous number of experiments (12 figures, many of them composite figures with several panels, plus 6 supplementary figures). We strongly believe that it is not necessary to determine the half-lives of the *trxA* transcripts because ALL experiments (reporter fusions, structure probing, toeprinting) unambiguously support translational control.

4. Please add a Northern blot that would show the difference in the expression ratio between the long and short endogenous isoforms of trxA.

Because we show very convincing RNA-seq data on the ratio of the long and short isoforms, an additional experiment is not necessary. This result was somewhat hidden in Fig. S6 in the original manuscript because it was only briefly mentioned in the Discussion. We now mention this important result earlier in the Results section and accordingly moved the figure forward as Fig. S1.

5. Figure 5B. I understand the interest of using trxA Rep as a control for the toeprint experiment because of its inability to respond to temperature. However, this experiment would be better with a control WT mRNA that does not exhibit RNAT capacities like yopN for example.

We are bit confused by this comment and the suggestion to use yopN as a control that "does not exhibit RNAT capacities". Actually, yopN is one of the best performing RNATs in *Yersinia* and we used it as positive control many times (Pienkoß et al. PLoS Pathogens, 2021).

We prefer to use repressed versions because derepressed versions are more difficult to design. Sometimes, they adopt alternative structures (Fig. 9A), or they still behave in a temperature-responsive fashion because parts of an extended structure are maintained (e.g., Pienkoß, Javadi et al. J Mol Biol 2022).

6. While sodC is relatively constant is terms of activation through temperature, the Ecoli sodB was activated 2.5X whereas Yersinia sodB mRNA was activated 1.6X by temperature in Fig 3. This is quite different from Fig 6, where Ecoli sodB is activated 5.2X and Yersinia is activated 1.0X. Please discuss this difference.

In contrast to ratiometric approaches like the roGFP2 system, beta-galactosidase experiments suffer from a certain degree of day-to-day fluctuations. This depends on various parameters, in particular freshly prepared solutions like the inducer arabinose or the substrate ONPG, but also on differences in the growth phases when samples were taken. Despite such differences, the results are very consistent in showing that *sodB* does not act in the RNAT-like fashion in *Yersinia*.

We assume that when saying "*Ecoli sodB*", the reviewer means *Yersinia sodB* measured in *E. coli*.

7. Figures 4 and 6, as well as 5 and 7, could be fused together since they use pretty much the same approach and obtained similar results. The data could be summarized into a table so that we could compare the different genes more easily.

As mentioned above in response to comment (3), the manuscript already contains many composite figures. We considered fusing figure 4 with 6 and figure 5 with 7 but combining two already complex figures would make them even bigger. In the interest of comprehensible RNA structure information in the (A) panels, we decided against reducing the size of the figures to squeeze more information into large figures that would cover an entire page.

8. Figure 12. The fluorescence intensity should use a different color than yellow as it is relatively harder to compare with higher contrast colors.

We agree and have adjusted the colors.

9. I would move the first paragraph of the Discussion in the Introduction. As written, this paragraph is more part of a wider introduction of the subject instead of focusing on the most important results of the work.

After some consideration, we decided to keep at least a short intro into the Discussion to set the stage for the further considerations. Given the extensive length of the manuscript, however, we tried to avoid redundancies and streamlined the paragraph.

Reviewer #2:

In this manuscript, the authors investigated .....
This was a very well written manuscript that clearly communicated the author's rationale, hypotheses, experimental design, and results. All of the experiments included appropriate controls, and the interpretations of the results were justified by their data and previous reports in the literature. The findings significantly improve our understanding of the role of thermosensing RNAT structures in the adaptation of Y. pseudotuberculosis to the mammalian host environment. I enjoyed reading this manuscript and have no significant critiques.

Thank you! We enjoyed this positive judgement.

Reviewer #3:

This manuscript characterizes an important regulatory link between temperature sensing and the oxidative stress response in the food borne pathogen Y. pseudotuberculosis… …
This work is a follow-up of the authors' previous study comparing the in vitro RNA structurome of Y. pseudotuberculosis at 37˚C vs 25˚C, which suggested possible RNATs in the oxidative stress genes. The in vitro characterization of the putative RNATs, including RNA structure probing and toeprinting, are solid. However, as detailed below, the manuscript lacks strong evidence supporting their in vivo function. 1) What is the structural difference of the proposed RNATs at 25˚C vs 37˚C in Yersinia? 2) Is the RNAT-mediated translational regulation important for oxidative stress response? These questions need to be addressed before publication.

Thank you for the positive judgement of our work and the constructive questions. For responses, see below.

Major points:
1) Do the proposed RNATs exhibit different structures in Yersinia at 25˚C vs 37˚C? The authors need to provide evidence showing the RNA structural change in vivo, e.g., based on data from (Twittenhoff et al., 2020). It is also unclear how the PARS-derived RNA structures shown in Fig. 4, 6, 8, 9 are predicted. Are they based on their previous publication (Righetti et al., 2016)? Are these RNA structures at 37˚C or 25˚C? The authors should cite the reference and explain how the structure model is predicted in the Material and Methods.

We agree that it would be nice to correlate the data from our present study to the *in vivo* RNA structurome data published by us in 2020 (Twittenhoff, Brandenburg, Righetti et al., Nucleic Acids Res). Unfortunately, that is not possible because the ROS defense genes are not highly expressed in *Yersinia*. Therefore, we do not have available a sufficient read depths to extract robust structural information. For example: Consistent with transcriptional control of *katY* (Fig. 2 in our manuscript), we have few reads on *katY* at 25°C. Several hundred read counts at 37°C suggest more transcript at higher temperature but are not yet sufficient to calculate a reliable RNA structure. For this, one would need several thousand counts covering the entire transcript.

In the future, a recently established improved Lead-Seq protocol (called Led-Seq; Kolberg et al., Nucleic Acids Res, 2023) might be suitable to solve this bottleneck.

Yes, the structures shown are based on our PARS publication (Righetti et al, PNAS, 2016). In most cases, they are the 25°C structures unless minimal fold energy (MFE) structures, the structure

probing experiments shown in this manuscript, and/or site-directed mutagenesis suggested that the 37°C structure was more likely. This information is now provided in the figure legends.

2) The in vivo function of putative RNATs seems rather weak in Yersinia. The authors characterized the RNATs in sodB, sodC, katA, katY and trxA-short transcripts, but only katY (and slightly for trxA-s and sodC) showed a significant temperature responsive translation increase in Yersinia, compared to the negative control sodA (Fig. 3C). The authors should discuss why the katY RNAT is more temperature sensitive than the others.

We are still intrigued by the finding that the newly discovered *katY* thermometer is among the best performing RNATs ever. Although we and others are working on RNATs for many years, it is surprising that functional RNATs remain difficult to predict and design. Hence, we can only speculate why the *katY* thermometer is so efficient. It contains several unique features. Unless most RNATs that have their "anti-SD" sequence upstream of the SD sequence (*yopN*, for example), here it is downstream (Fig. 9A). Another unusual feature is that the SD sequence is paired by three strong GC bonds. On the contrary, the structure contains large bulges and loops, which might compensate for the strong binding and make the overall structure very temperature responsive. This might be the regions from where heat-induced melting starts as it was shown by NMR studies on other RNATs (Chowdhury et al., EMBO J, 2006; Rinnenthal et al., Nucleic Acids Res, 2010).

For the other genes besides katY, it is unclear how much the proposed RNATs affect their endogenous translation in Yersinia. Furthermore, the authors did not show whether these genes and their temperature sensitive regulation affect the oxidative stress response of Yersinia. Without in vivo evidence, the function of these RNATs is questionable.

Yes, the *in vivo* functionality of the *sod* 5'UTRs (functional *in vitro* and in *E. coli* but not in *Yersinia*) is questionable, and we never claimed that they are functional in *Yersinia*. Already in the Abstract, we state that these dynamic structures are presumably partially open in the living cell, and we added another conclusion to that effect in the Discussion.

The motivation of this study was to check if and which of these RNAT candidates are physiologically relevant. To this end, we systematically assayed transcription and translation of the ROS defense genes in an enormous, at least three-years effort. The answer to this question was that the short *trxA* transcript and the *katY* transcript, which was discovered in the context of this analysis, contain RNATs that are functional *in vitro*, in *E. coli* and – most importantly of course – in *Yersinia*. To address the physiological role, we show that the *katY* thermometer is relevant for protection under ROS stress at elevated temperatures. In our opinion, all these conclusions are well supported by the data presented in this manuscript.

3) The authors use stabilizing mutations of the proposed RNATs to examine the loss of temperature responsive regulation. However, all these mutants almost completely abolish translation of the reporter, making it difficult to interpret the results. A likely better way is introducing destabilizing base changes to the stem-loop in RNATs (but without affecting the SD sequence) to test the reduced translation inhibition at 25˚C. Additionally, a complementary mutation that recovers the base-pairing is expected to restore the RNAT function and the temperature-sensitive translation control, which would support the regulatory role of RNA structure rather than sequence.

For the most relevant RNAT in the context of this study, namely *katY*, we used both destabilizing and stabilizing mutations (Fig. 9).

The reviewer already pointed out the most critical caveat of mutant construction. The SD sequence must not be affected! Hence, the available space for the design of point mutations is limited. The construction of destabilized version is often hampered by the fact that the derepressed sequences

tend to adopt alternative structures (see Fig. 9A as an example), which complicates the interpretation of the results. Hence, repressed versions are typically more straightforward to design.

Complementary or compensatory mutants that would recover base-pairing and restore RNAT function are almost impossible to construct because one would have to mutate the SD sequence or regions immediately flanking the SD sequence. Doing so, would interfere with translation initiation.

4) The temperature regulated KatAY expression affects the susceptibility of Yersinia against H2O2. However, it is unclear how much the RNAT-mediated translation regulation contributes to it, as the mRNA level of katY is already 30-fold higher at 37˚C than at 25˚C. In addition to WT and katA/Y deletion (Fig. 10, 12), comparison to the mutants with nonfunctional RNATs (e.g., those mentioned in 3)) would significantly strengthen the conclusion.

Adding up the experimental data from transcriptional and translational measurements does not necessarily reflect the natural situation. Our results show that the RNAT-mediated translational control has a dominant effect on *katY* expression. No matter how strong transcription is enforced (from an arabinose-inducible plasmid in Fig. S4), KatY is produced almost exclusively at 37°C demonstrating that the thermometer efficiently silences translation at 25°C regardless how much transcript is present. New drawings in revised Fig. 11 and S4 depict the different experimental set-ups, and a new statement in the revised Discussion re-emphasizes this important finding.

We measured several other mutants and mutant combinations not mentioned in the already very dense manuscript. All results show that *katY* is the major player in temperature-inducible ROS protection. Some of the results are shown below.

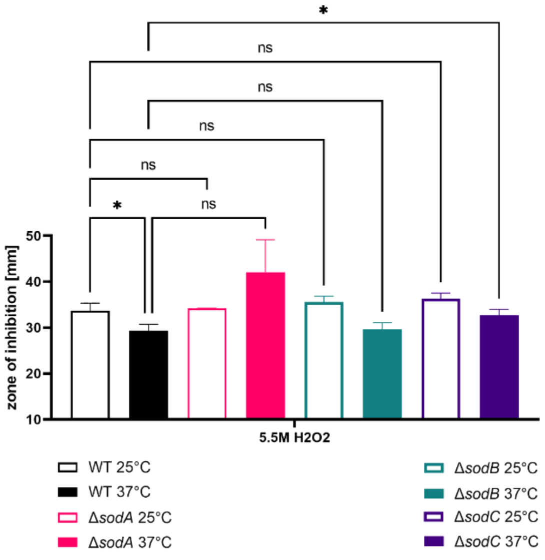

Disk diffusion assay for H_2O_2 was conducted by applying 3 µl of 5.5 M H_2O_2 onto paper disks on soft agar containing a bacterial suspension. After 24 h of growth at the indicated temperature the zone of inhibition was measured. The experiment was carried out multiple times and each time in biological triplicates and two technical replicates. Asterisks indicate statistically significant differences by oneway ANOVA (n = 3; * p < 0.05; ** p < 0.01; *** p < 0.001; ns, not significant)

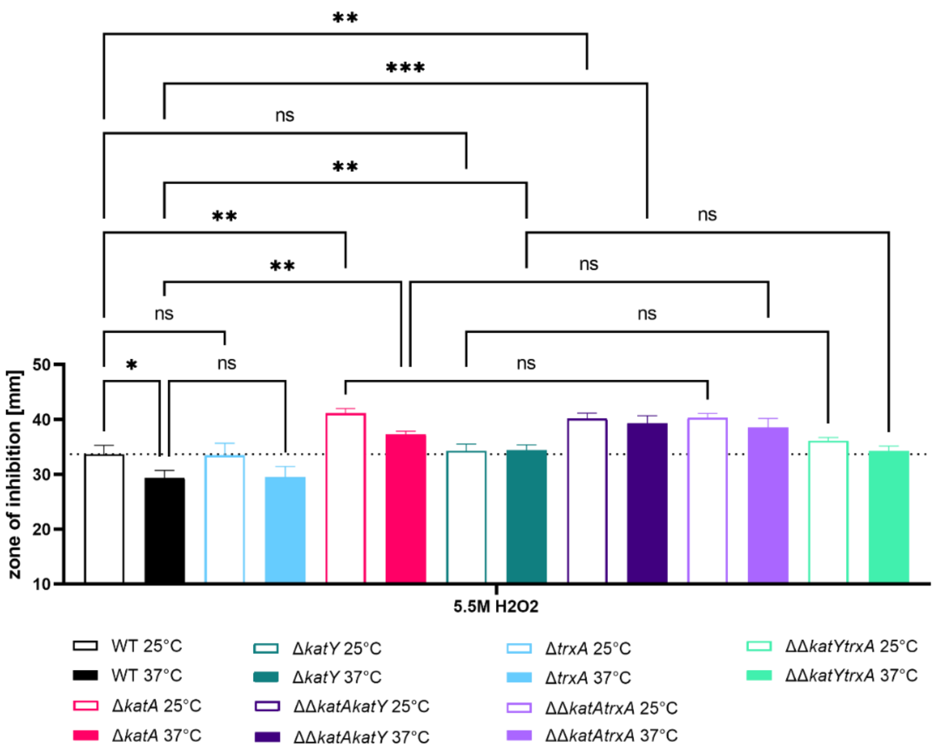

Disk diffusion assay for H_2O_2 was conducted by applying 3 µl of 5.5 M H_2O_2 onto paper disks on soft agar containing a bacterial suspension. After 24 h of growth at the indicated temperature the zone of inhibition was measured. The experiment was carried out multiple times and each time in biological triplicates and two technical replicates. Asterisks indicate statistically significant differences by oneway ANOVA (n = 3; * $p < 0.05$; ** $p < 0.01$; *** $p < 0.001$; ns, not significant)

5) The overall temperature responsive induction of genes does not reflect the combination of transcriptional and translational regulation. KatY has both its mRNA level and translation level increased ~30-fold at 37˚C vs 25˚C, measured by RNA-seq and translation reporter, respectively. However, the western blot shows only 17-fold induction (Fig. 11). The quantification of KatA induction has similar issues. The authors should address these discrepancies between different methodologies.

See response to the comment above.

6) Results do not agree between figures/panels. For example, western blots in Fig. 3C (sodA, sodB, sodC) and Fig. 6C (sodC in E. coli, sodB, sodC in Yersinia) do not agree with the beta-galactosidase activity for the translational fusion reporters. The beta-galactosidase activity results of the same translational reporter are different between figures (e.g., sodB reporter in E. coli in Fig. 3B vs Fig. 6C). If different controls/adjustments were used, it should be specified in the figure legend.

See response to comment (6) from reviewer 1.

Additional points:
1) Fig. 2 does not show replicates or statistical comparison of the RNA-seq and qRT-PCR data. Some data bars are missing – it is unclear whether the data is not available or is zero.

The figure legend has been revised to include more information. Furthermore, Table S1 was extended.

2) Comparison of mRNA level is missing for translational reporters shown in Fig. 4, 6, and 8.

Since the results did not provide evidence for these candidates being functional RNATs in *Yersinia*, we did not invest in additional experiments.

3) In Fig. 10A and C, the katY deletion has higher catalase activity at 37˚C than 25˚C, but the inhibition zone of H2O2 does not show a significant difference. In general, the liquid growth (in Fig. S1) seems more sensitive than the disk diffusion assay for measuring H2O2 detoxification. The authors might consider replace the disk diffusion results with the Fig. S1.

We considered swapping parts of Fig. 10 and Fig. S1 (now Fig. S2) but decided against it due to space constraints. We agree that it is useful to look at both the disk diffusion assay and the growth experiment.

4) In Fig. S3, why is delta katA + A more sensitive to H2O2 at 37˚C than 25˚C?

Maybe, the His-tag is slightly inferring with KatA activity at elevated temperature. The most important result is that some complementation was observed and that the strain does not behave like the *katA* mutant. As with many complementation experiments in the literature, a complete restoration of the WT phenotype was not necessarily expected.

5) The protein abundance of KatY is much higher than that of KatA at 37˚C (Fig. 11). Why does KatA still play a more important role in H2O2 detoxification?

This is what we asked ourselves for a while before we measured the catalase activities. Apparently, KatA is a much "better" catalase than KatY (Fig. 10 C and S2B, which now is S3B). This is consistent with reference 20 (Wan et al., Front Microbiol 2021).

6) Table S1 does not include all the data shown in Fig. 2.

It now does in the revised version.

7) The authors use "anti-SD sequence" for the region that base-pairs with the SD sequence. It can be confusing because anti-SD usually refers to the 3' end of 16S ribosomal RNA. The authors may specify its definition in the text.

We agree that the term "anti-SD sequence" can be confusing. We considered anti-RBS sequence but decided against it because for all the RNATs shown in this study it is in fact the SD sequence that is blocked by an "anti-SD sequence". For lack of a better designation, we now define the meaning in the text when we first use it.
